# Cardiac electrophysiological adaptations in the equine athlete—Restitution analysis of electrocardiographic features

**DOI:** 10.1371/journal.pone.0194008

**Published:** 2018-03-09

**Authors:** Mengye Li, Karan R. Chadda, Gareth D. K. Matthews, Celia M. Marr, Christopher L.-H. Huang, Kamalan Jeevaratnam

**Affiliations:** 1 Physiological Laboratory, University of Cambridge, Cambridge, United Kingdom; 2 Faculty of Health and Medical Sciences, University of Surrey, Guildford, United Kingdom; 3 Rossdales Equine Hospital and Diagnostic Centre, Exning, Suffolk, United Kingdom; 4 Division of Cardiovascular Biology, Department of Biochemistry, University of Cambridge, Cambridge, United Kingdom; Georgia State University, UNITED STATES

## Abstract

Exercising horses uniquely accommodate 7–8-fold increases in heart rate (HR). The present experiments for the first time analysed the related adaptations in action potential (AP) restitution properties recorded by in vivo telemetric electrocardiography from Thoroughbred horses. The horses were subjected to a period of acceleration from walk to canter. The QRS durations, and QT and TQ intervals yielded AP conduction velocities, AP durations (APDs) and diastolic intervals respectively. From these, indices of active, *λ* = QT/(QRS duration), and resting, *λ*_0_ = TQ/(QRS duration), AP wavelengths were calculated. Critical values of QT and TQ intervals, and of *λ* and *λ*_0_ at which plots of these respective pairs of functions showed unity slope, were obtained. These were reduced by 38.9±2.7% and 86.2±1.8%, and 34.1±3.3% and 85.9±1.2%, relative to their resting values respectively. The changes in *λ* were attributable to falls in QT interval rather than QRS duration. These findings both suggested large differences between the corresponding critical (129.1±10.8 or 117.4±5.6 bpm respectively) and baseline HRs (32.9±2.1 (n = 7) bpm). These restitution analyses thus separately identified concordant parameters whose adaptations ensure the wide range of HRs over which electrophysiological activation takes place in an absence of heart block or arrhythmias in equine hearts. Since the horse is amenable to this in vivo electrophysiological analysis and displays a unique wide range of heart rates, it could be a novel cardiac electrophysiology animal model for the study of sudden cardiac death in human athletes.

## Introduction

It is widely established that exercise improves health outcomes, such as by reducing the burden of cardiovascular disease [1]. However, exercise has paradoxically been associated with an increased risk for cardiac events in a small number of individuals [2]. In particular, the highest risk for exercise-related sudden cardiac death (SCD) occurs in athletes with quiescent cardiovascular defects [3]. In athletes, SCD remains the most common medical cause for sudden death and its annual estimated incidence rate is between 2.3 to 4.4/100,000 persons in the USA [2, 4–6]. Furthermore, veteran athletes are more likely to require the fitting of an electronic heart pacemaker later in life [7–9]. Despite these clear epidemiological associations for arrhythmic tendency in athletes, the underlying electrophysiological mechanisms are not well understood.

Previous studies have successfully exploited murine models to investigate the association between chronic exercise and arrhythmogenic mechanisms. One study showed that exercise-induced remodeling of the sinus node occurs in murine models with a downregulation of HCN4 mRNA and protein, causing a decrease in *I*_f_ and subsequent sinus bradycardia [10]. Other studies in mice [11] and rats [12] have demonstrated increased cardiac fibrosis and arrhythmia inducibility after chronic intensive exercise. However, the limitations of murine models constrain the translational inferences that can be made from such studies.

Nevertheless, the horse as an animal model could represent a novel system to investigate SCD in athletes with advantages over the classic murine models. Firstly, from an athletic life course perspective, horses, but not mice or rats, undergo a similar sequence to humans involving training, peak performances, and retirement from active participation. This allows investigation into underlying electrophysiological changes in the veteran athlete. Secondly, murine resting heart rates (HRs) range from 500 to 700 beats per minute (bpm), 10-fold higher than resting human HRs. During exercise, murine HRs can increase ~10 to 50% whereas human HRs can increase by up to 300% [13]. On the other hand, equine heart rates follow a more similar pattern to humans, ranging from as low as 20bpm to ~229bpm during maximum exertion [14]. Thus, murine models display a limited incremental range of heart rates compared to the horse, making the horse more amenable to physiological analysis of exercise-induced heart rate changes. Thirdly, human repolarization is attributable to different ionic currents to murine models whereas initial reports indicate that horse repolarizing currents have similarities to humans [15, 16]. Finally, amongst veterinary species, horse hearts have a unique ability of displaying spontaneous atrial fibrillation (AF) in the absence of gross structural abnormalities, as occurs in humans.

Several studies have investigated the prevalence of such arrhythmias in horses. Thus, in a multicentre international study, gross and histological cardiac lesions accounted for only 54% of sudden cardiac death cases in Thoroughbred horses, leaving a possibility of fatal cardiac arrhythmia in the remaining cases [17]. Electrocardiographic (ECG) studies demonstrated high prevalences of both premature depolarization [18] and arrhythmias [19], including AF [20], in apparently healthy Thoroughbreds. However, our limited understanding of equine cardiac electrophysiology compromises development of reliable diagnostic, therapeutic or prognostic strategies [21–25]. This lack of understanding also prevents the equine athlete from being explored as a model for understanding physiological and pathological cardiac electrophysiology in the human athlete.

In order to validate the equine athlete as an electrophysiological model for human athletes, it is important to determine whether the equine system is amenable to standard electrophysiological analysis techniques at incremental heart rates. Previous in vitro studies had investigated conditions under which incremental increases in HR were associated with arrhythmic substrate in isolated, denervated, Langendorff-perfused murine [26–29], guinea pig [30, 31], and porcine hearts [32]. They explored changes in AP duration (APD) and conduction velocity (CV) at progressively increasing HRs. They then derived restitution curves plotting the dependence of these recovery and propagation variables upon the magnitudes of the diastolic intervals (DI) separating successive APs [26, 28, 33, 34].

A more complete analysis then went on to incorporate terms representing AP propagation by plotting the wavelengths of active *λ* and resting *λ*_0_ myocardial regions involved in the excitation process [27]. The *λ* term thus represents the length of tissue that is depolarized by the AP wavefront at any one time, and the *λ*_*0*_ term is the length of repolarized tissue that trails behind the depolarizing tissue. The sum of these two, active, λ, and resting wavelength, λ_0_, terms gives the basic cycle distance (BCD); thus: BCD = λ + λ_0_. This is the distance that separates the depolarizing wavefronts of two successive APs propagating through the myocardial tissue. The variation of these terms with changes in HR and conduction velocity provides a basis for the quantitative derivation of critical conditions at which ordered electrophysiological excitation breaks down into AP alternans, thereby generating arrhythmic substrate [35].

Thus, previous cellular work has shown the importance of restitution analysis in various animals from first principles. Nevertheless, there remains a need to conduct such analysis at a systems level with intact autonomic innervations in an animal model that is more translational to the human athlete. The present paper accomplished such in vivo restitution analyses on the equine heart for the first time using Thoroughbred racing horses in training. Non-invasive electrocardiographic (ECG) recordings provided measurements of the QRS duration, QT interval, and TQ interval. These provided indications of CV, APD and DI respectively. This made it possible to assess the HRs over which stable ventricular activation could be predicted without an appearance of arrhythmic substrate.

## Methods

No standard ethical approval was required as animals were not specifically used for the sole purpose of this retrospective study. All data was collected during routine clinical work up and have been acquired non-invasively. Additionally, all retrospective data was anonymized prior to analysis. A total of 34 ECGs recorded from healthy Thoroughbred horses in race training presented for workups at Rossdales Equine Hospital and Diagnostic Centre (Newmarket, Suffolk, United Kingdom) were screened retrospectively for possible further study. ECGs had been recorded while horses were exercised as part of their established performance assessment programs. All horses were of racing age (2–8 years) at the time of testing. None showed clinically significant cardiac abnormalities on prior routine cardiovascular examination. Twenty-three were males (13 geldings and 10 colts) and 11 were females. Each horse was atraumatically fitted with telemetric electrocardiographic (ECG) recording equipment. ECG recordings were then acquired before and during a period of acceleration from walk to canter. The protocol yielded a systematic series of recordings at a range of relatively steady incremental heart rates. This emulated incremental pacing protocols that have previously been applied in studies of cardiac function in vitro [26–28]. The ECG data acquired in each horse was exported and further signal processing and analysis was performed. Only ECG complexes that were clearly discernible were analysed. Data from 7 horses spanning between 10–18 min of ECG recording fulfilled the following necessary criteria for full quantitative analysis: (1) Data points obtainable over the entire range of basic cycle lengths (BCLs), giving HRs from 22 bpm to the 7-fold greater frequency of ~181 bpm and (2) Scatter in the data points consistent with testing against established restitution functions.

**Fig 1:**
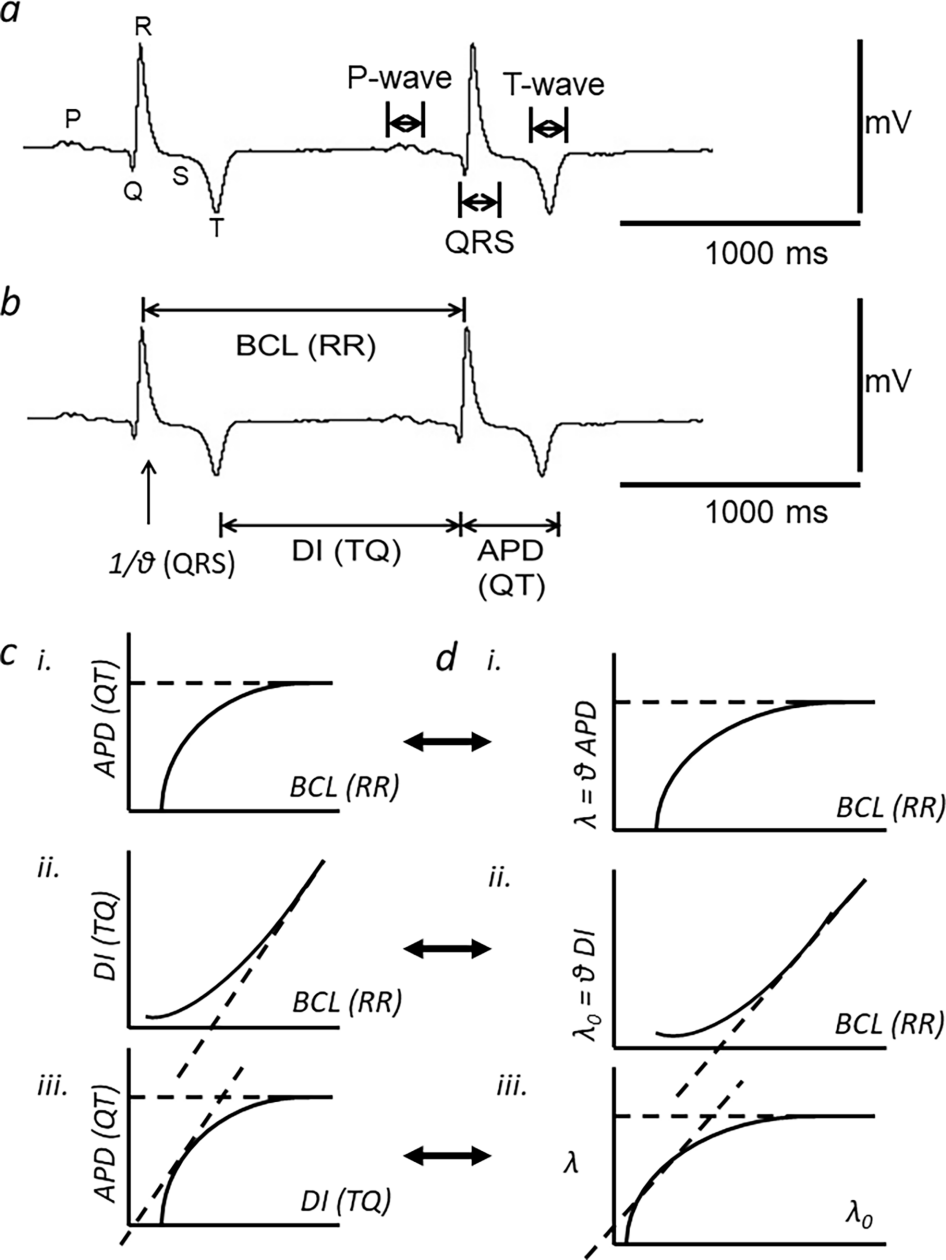
Determination of electrographic intervals from equine ECG recordings and restitution analysis in the temporal and spatial domains. a) A typical equine ECG trace at a low heart rate (HR) of between 43–45 beats per minute (bpm). The P-wave, QRS-complex, and T-wave are annotated. b) The same trace showing the intervals RR, QRS, TQ and QT that the signal analysis yielded. These gave basic cycle length (BCL), conduction velocity (θ), diastolic interval (DI) and action potential duration (APD) respectively (Horse Eq46: age 7 years, thoroughbred female). c) Temporal patterns of action potentials (AP) over time: with progressively shortened basic cycle lengths (BCL) (i), with (continuous line) or without variations in APD (dashed lines) results in a corresponding dependence of diastolic interval (DI) upon BCL (ii), a unity gradient of the resulting APD(DI) restitution function (dashed tangent) provides an instability criterion relating AP recovery characteristics to alternans (iii). d) Analysis incorporating spatial AP propagation at velocity θ, generates active and resting wavelengths, λ and λ_0_, each varying with BCL (i and ii respectively). Unity gradients in the derived λ(λ_0_) plots (iii; dashed tangent) yield instability conditions that additionally incorporate AP propagation contributions. (c and d adapted from [38]).

Fig 1A and 1B exemplify determination of ECG intervals from a typical record obtained near the beginning of the experimental protocol. Previous reports have identified ECG deflections with the first derivative of the corresponding atrial or ventricular action potential waveforms [36]. Thus, the P-wave was the initial deflection in the ECG trace, identified with atrial activation. It could be either monophasic or biphasic in waveform. The Q-wave was defined as the trough of the subsequent sharp downward deflection. There then followed the R-wave, determined as the maximum point on each PQRST complex. This was followed by a return of the trace to the isoelectric line identified as the S-wave. The QRS deflections have been attributed to the generation and propagation of ventricular depolarisation through the cardiac conducting system and contractile myocytes. Using these points, it was then possible to obtain the QRS duration, and the reciprocal of which, 1/(QRS duration), reflected the ventricular conduction velocity, denoted by θ in this study. Each ECG complex ended with a typically negative T-wave deflection. The trough of the T wave was defined as the point at which the minimum value was attained in each PQRST complex. The subsequent end of the T-wave was identified with a return of the trace to the isoelectric line with zero slope. This would correspond to full recovery of the action potential, with the resulting QT interval therefore providing an index of the ventricular action potential duration [36]. In human clinical measurements it is thus used in preference to the time taken to the peak or trough of the T wave [37]. In the present studies, it could be consistently determined by quantitative analysis of the digital signals. This procedure identified the relevant region of the electrocardiographic complex by identifying the T wave trough as a reference marker. The end of the T wave would then fall between this trough and the time at which the trace had rejoined the iso-electrical line. From the trough, the cursor was used to determine the slope of the tangent to the trace at successively later time points. It was thus possible to determine the time at which the tangent had reached a zero slope that was identical to that of the iso-electric line. This procedure yielded consistent values of QT interval, or where alternans was observed, alternations in these values that nevertheless were transient and followed by resumption of constant QT intervals. Measurement of the RR interval between each given and the succeeding PQRST complex provided the range of BCLs (BCL in seconds = 1/(RR interval) and the HRs (HR in bpm = (1/BCL) × 60). The TQ (i.e. diastolic) interval preceding the QRS complex under examination was derived from the preceding RR and QT intervals (TQ interval = RR interval–QT interval). We then derived previously established indices of active, *λ* = QT/(QRS duration), and resting, *λ*_*0*_ = TQ/(QRS duration), ventricular AP wavelength in these horses.

These indices and the wide range of BCLs shown by the horse allowed a restitution analysis to be performed for the first time in an intact animal, in both the temporal and spatial domains (Fig 1C and 1D). Thus, these example figures summarize the entire experimental design concept and each panel is represented by corresponding experimental data. Data is expressed in the form of mean ± SEMs. All statistical comparisons are made between two parametric data sets derived from the same horse. All data sets are expected to follow a normal distribution; thus, the student’s T-test was employed to assess for statistically significant differences between data sets. The mathematical functions were fitted to observed data using standard plotting and least-squares fitting algorithms (Origin, Microcal, Calif. USA).

## Results

### Accommodation of ECG waveform to increasing HRs

**Fig 2:**
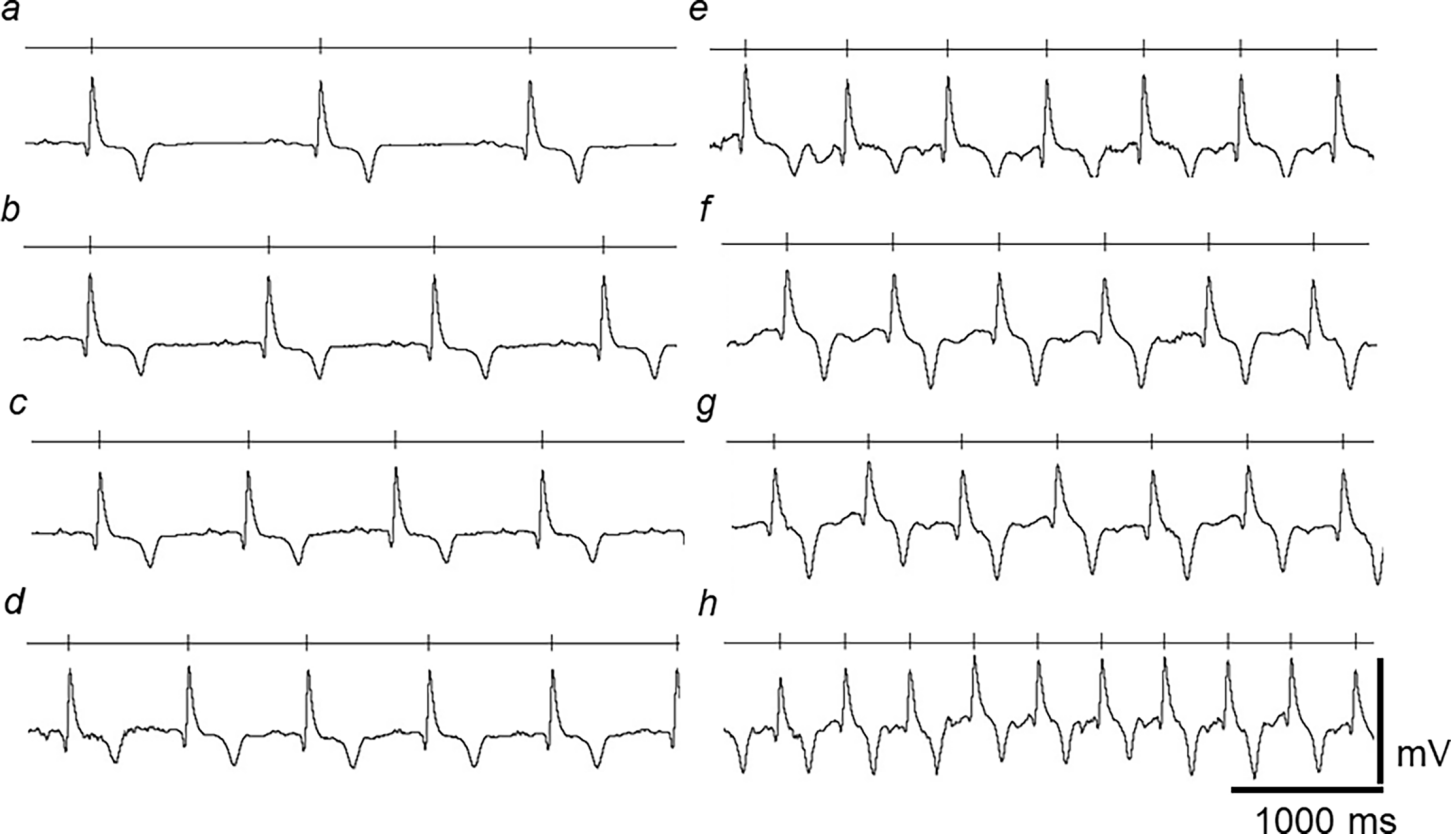
Accommodation of ECG waveform to increasing HRs. ECG traces obtained through a range of low to high HRs covering the normal physiological spectrum encountered in horses. Each trace is displayed as a rhythm strip; the vertical markers on the horizontal line above trace marks the timing of successive R-waves. The range of HRs within each of trace was (a) 40–45 (b) 51–55 (c) 61–62 (d) 73–76 (e) 89–93 (f) 105–108, (g) 118–120 and (h) 140–145 bpm. (Same horse as shown in Fig 1).

Fig 2 illustrates typical ECG records at different heart rates (HRs) obtained from an experimental run. The vertical marker drawn through the lines above each record mark the timing of successive R-waves in the ECG wave, giving HRs of (a) 40–45 (b) 51–55 (c) 61–62 (d) 73–76 (e) 89–93 (f) 105–108, (g) 118–120 and (h) 140–145 bpm. Normal PQRST complexes were observed at all HRs studied. However, the ECG waveforms showed evidence of accommodation to increasing HRs and their consequent falls in BCL. This permitted a greater number of action potentials (AP) to be completed within the same time interval. Despite the reduced cycle lengths, the magnitude of successive ECG signals, reflected in the height of the QRS complex, remained relatively constant throughout, suggesting continued and stable contact between leads and ECG recording system throughout the experimental run.

### Presence of transient alternans in all horses at both low and high HRs

**Fig 3:**
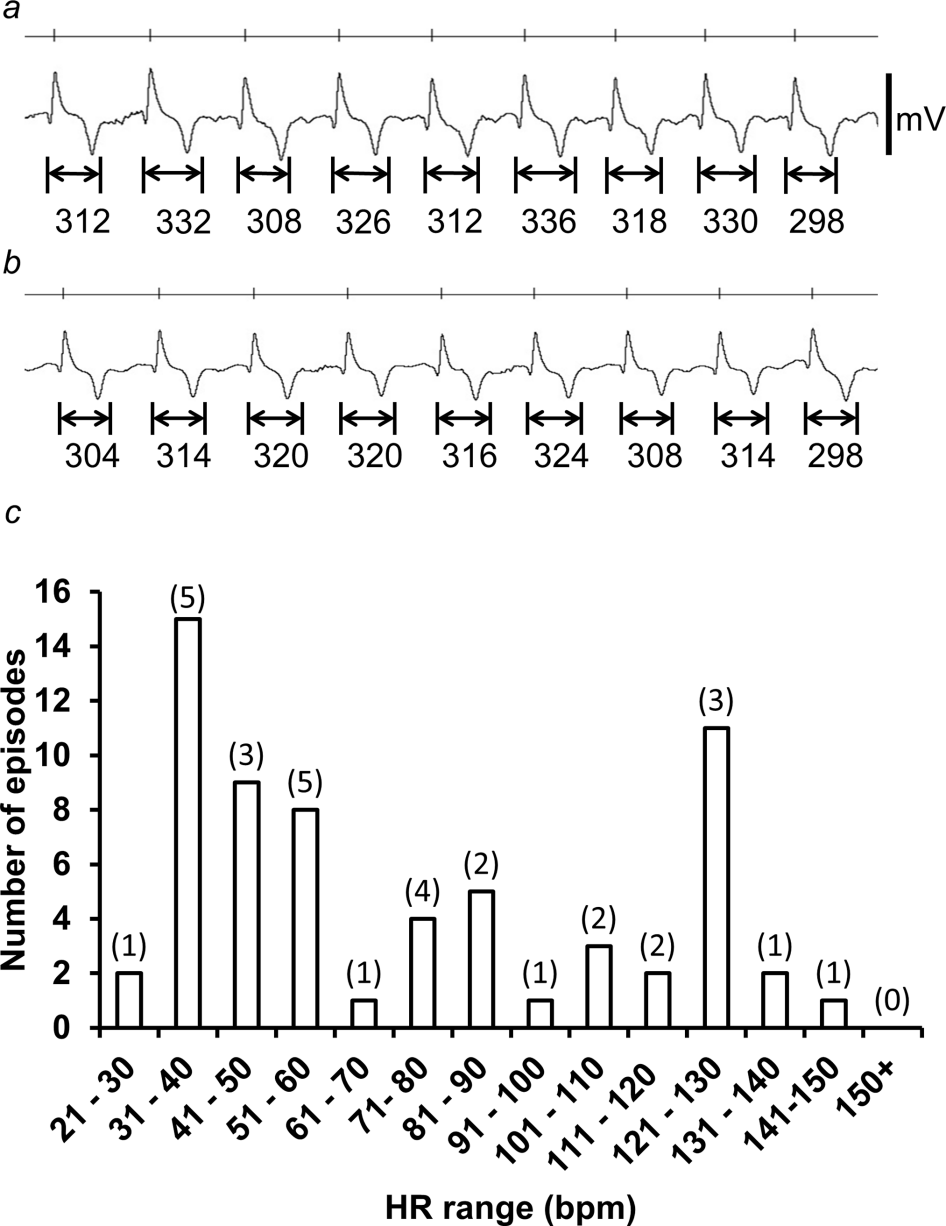
Presence of transient alternans in all horses at both low and high HRs. (a) Transient alternans recorded at a HR over the range of 103–113 bpm. The trace was taken from an episode of transient alternan, which lasted a total of 14 beats (7.8 secs in duration). (b) A trace recorded from the same horse, in absence of alternans, and over a similar HR, ranging from 105–109 bpm. The QT interval for each PQRST complex is marked below the trace, demonstrating alternations in the QT interval between successive complexes. The vertical markers above each trace mark the timings of consecutive R-waves of each PQRST complex. (c) A histogram showing the number of episodes of transient alternans recorded within each interval HR of 10 bpm (value in brackets is the number of horses from which a HR within that interval was recorded). (Same horse as in Figs 1 and 2).

All the ECG traces examined quantitatively showed episodes of QT alternans. These episodes were identified as periods during which there were alterations in QT interval with a >8 ms difference between consecutive beats that extended over 6 or more beats. Fig 3A and 3B contrast ECG records observed in the presence (a) and the absence (b) of alternans, observed over similar ranges of HRs of 103–113 bpm (a) and 105–109 bpm (b) respectively. Traces were obtained from the same horse as shown in Figs 1 and 2.

Fig 3C summarizes the incidences of such episodes at different HRs, with the number of horses from which such HRs were observed denoted in brackets. Such episodes of alternans occurred more frequently at the lower HRs, particularly at HRs of between 31–60 bpm. These episodes of alternans were always transient, lasting an average of 7.1 ± 0.2 beats (7.2 ± 0.4 sec) (n = 64 episodes over 7 horses) with only 3 episodes in total extending to 12–14 beats. No episodes exceeded more than 14 beats or lasted longer than 17.5 sec. There were no alternans episodes that developed into observed arrhythmias. These findings contrast with previous reports that associated the onset of major ventricular arrhythmia with appearance of *persistent* alternans at the *highest* rates studied [27, 28, 33, 35].

### Dependence of basic and derived ECG parameters on BCL

**Fig 4:**
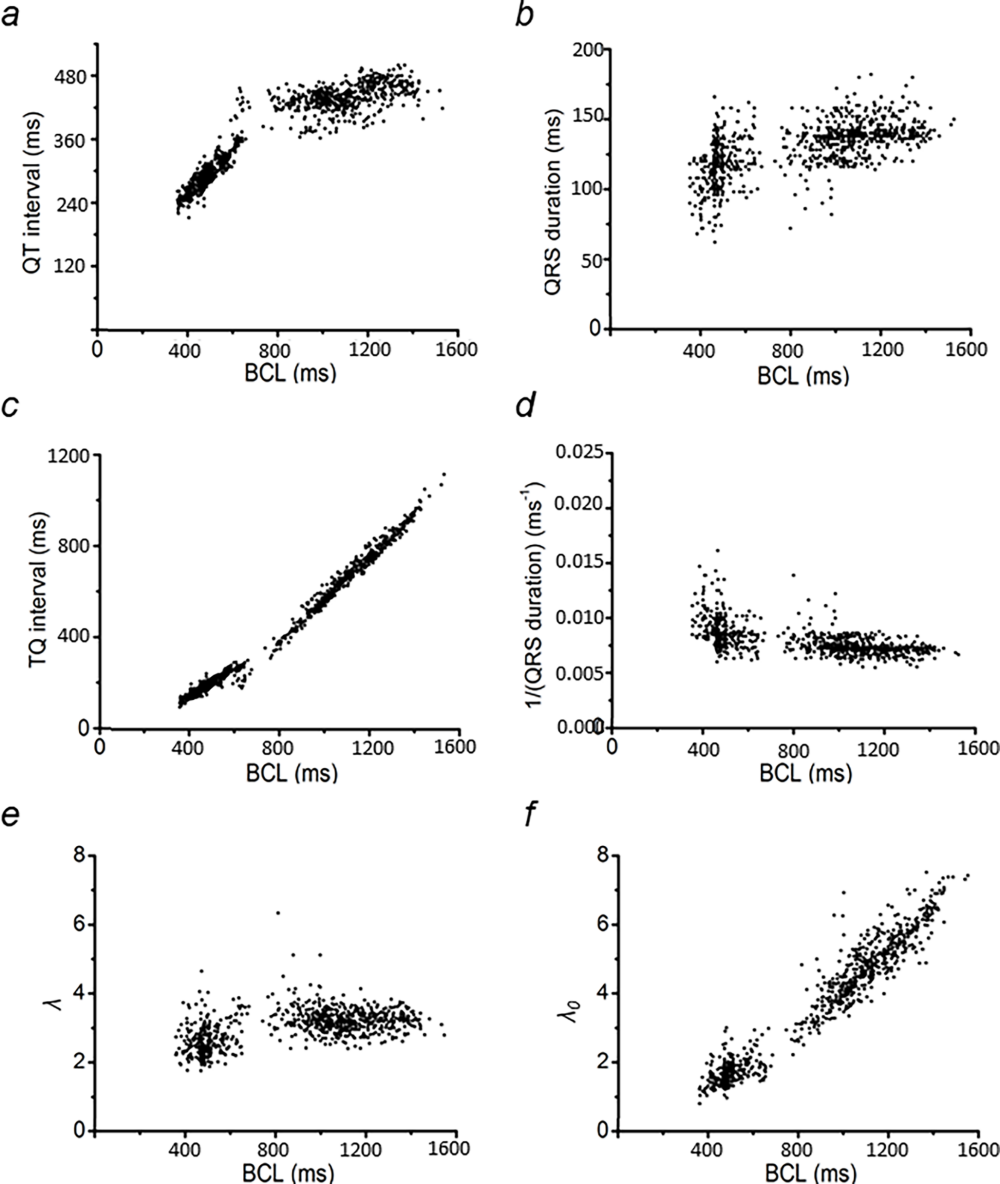
Dependence of basic and derived ECG parameters on BCL. The effect of increasing HR (depicted as a shortening of BCL) on the measured QT interval **(a)** and QRS duration **(b)**, and on the calculated TQ interval **(c)**, 1/(QRS duration) **(d)**, λ **(e)**, and λ_0_ **(f)** values. Data used were obtained from analysis of ECG recordings from the same horse as in Figs 1–3.

Fig 4 illustrates analysis of ECG waveforms in a typical data set at increasing HRs, expressed as BCLs. It plots **(a)** QT interval, **(b)** QRS duration, **(c)** TQ interval, and **(d)** 1/(QRS duration) against BCL. The values of *λ* and *λ*_*0*_ are represented by the dimensionless quantities λ = (QT interval)/(QRS duration) and *λ*_*0*_ = (TQ interval)/(QRS duration) respectively (Matthews et al., 2013). The relationships between the respective variables *λ* and *λ*_*0*_ with BCL are illustrated in Fig 3E and 3F respectively.

QT intervals were relatively stable at BCLs >800 ms, and fell at lower BCLs, corresponding to increases in HR. Correspondingly, the TQ intervals fell with decreasing BCL, but doing so to a lesser extent at BCL<800 ms. This reflects DI correspondingly decreasing with increasing HR. In contrast, QRS duration showed a decline, corresponding to a sharp increase in the conduction velocity term (1/(QRS duration)), and only at BCL< 600ms. Finally, *λ* only fell at BCL<600 ms, with *λ*_*0*_ correspondingly decreasing with decreasing BCL.

### Plots of QT interval and wavelength restitution

**Fig 5:**
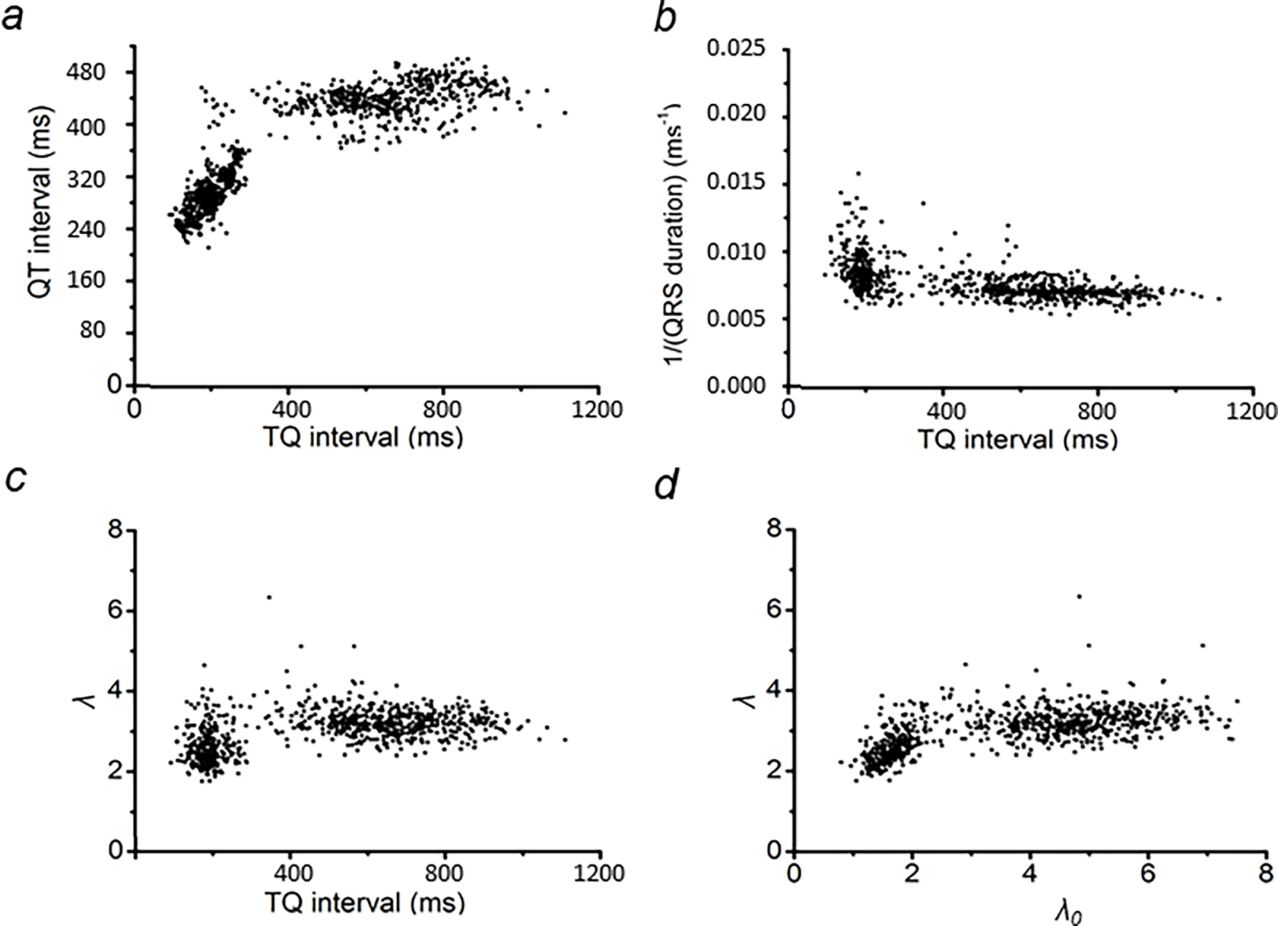
Representative figure for the analysis of QT interval and wavelength restitution plots. Plots of QT interval (a) and 1/(QRS duration) (b), and *λ*0 (c) against TQ interval, and *λ* against *λ*_0_ (d).

Fig 5 summarizes the initial analyses of the data from the representative horse displayed in Fig 4. It derives restitution plots of APD and of CV against DI [26] and of *λ* and *λ*_0_ [27]. In this analysis, APD was represented by QT interval, CV by the inverse of the QRS duration, and the DI preceding each AP by the TQ interval. Graphs in Fig 5A–5C plot DI as the independent variable, as performed previously [26]. They showed the expected decrease in DI with increasing HR. This shortens the time allowed for recovery of Na^+^ channel function from refractoriness, and therefore reduces CV and APD. Fig 5D employs *λ*_*0*_ as the independent variable in common with previous reports [27].

The plot of QT interval against the TQ interval (Fig 5A) was similar in form to that of previous restitution representations of APD against DI [26]. Thus, the plot assumed a plateau with a decline at low TQ intervals. Over the 7 horses studied, this occurred at TQ intervals of <444.3 ± 32.5 ms (n = 7). In contrast, plotting 1/(QRS interval) against TQ interval resulted in relatively constant values that then increased at significantly lower TQ intervals of <298.6 ± 23.5 ms (n = 7, P<0.01). This suggested an increase in CV of the AP at high HRs.

The readings shown in Fig 5A and 5B made it possible to derive the corresponding values of *λ* (Fig 5C), which showed its decline from plateau values at markedly lower TQ intervals than did the QT interval (Fig 5A). Finally, Fig 5D plots a wavelength restitution curve that maps *λ* against *λ*_*0*_. This gave a gradual decline in *λ* with falling *λ*_*0*_.

### Complementary dependencies of λ and λ_0_ on BCL

**Fig 6:**
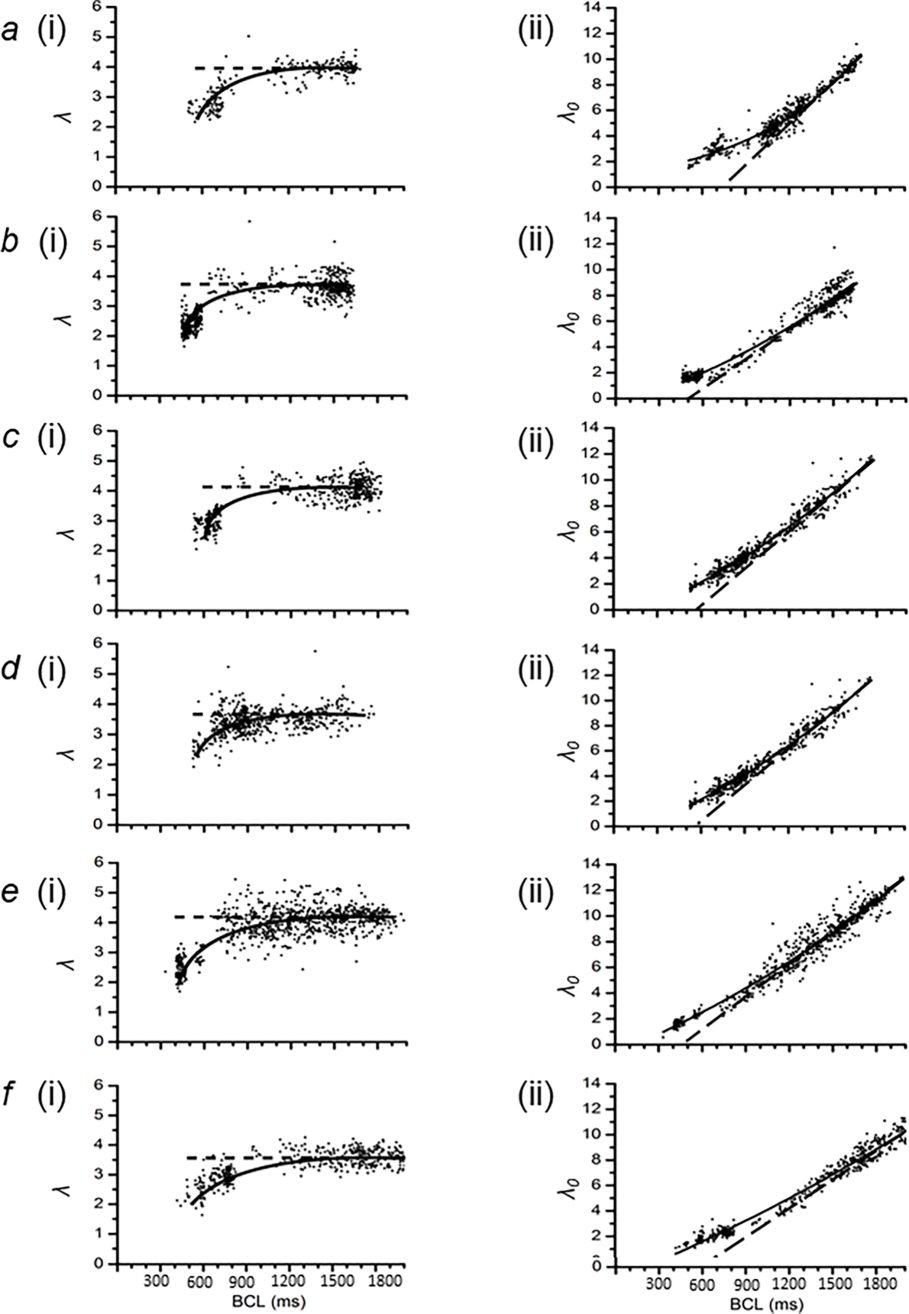
Complementary dependencies of λ and λ_0_ on BCL. (a)-(f). Plots of λ (i) and λ_0_ (ii) against BCL in six horses. The solid black line was manually fitted to demonstrate the trend of the data points. The dotted black line shows trends at the higher BCLs only. (a) Eq34 (a 2 year old male thoroughbred), (b) Eq39 (a 3 year old female thoroughbred), (c) Eq44 (a 4 year old female thoroughbred), (d) Eq52 (a 2 year old male thoroughbred), (e) Eq56 (a 2 year old male thoroughbred) and (f) Eq67 (a 3 year old male thoroughbred)).

Fig 6 show λ (i) and λ_0_ plots (ii) from six horses (a)-(f) in addition to the horse, which was used as an exemplar in Figs 1–5 to illustrate our analysis, providing a representation of the spatial extents of the active and resting regions of membrane at each BCL. The dotted black lines show trends at the higher BCLs. Those in (i) demonstrate the plateaus shown by the λ-BCL plots. Those in (ii) demonstrate the linear declines in λ_0_ with falling BCL. The solid black lines illustrate the trends in the experimental data through the entire range of BCLs studied. At the higher BCLs studied, the λ values showed constant values close to 3.8 ± 0.1 (n = 7) (Fig 6i). There was a corresponding value of λ_0_ of 7.2 ± 0.3 (n = 7) at BCLs of 1400 ms (Fig 6ii).

A decrease in BCL would correspond to a reduced basic cycle distance (BCD). The value of *λ* assumed a plateau and then declined at low values of BCL. The value of *λ*_*0*_ correspondingly showed a linear decline with declining BCL at the higher ranges of BCL, with similar slopes of (0.017 ± 0.001 (n = 7)) ms^-1^ between all horses. At low values of BCL, the points deviated from this linear relationship. This took place at a BCL that corresponded to the fall in *λ* from its plateau. Studies of the kind illustrated in Fig 4A and 4D attribute this adaptation of *λ* and therefore of *λ*_*0*_ with falling TQ intervals, to decreasing BCLs, a result of shortening of APD at high HRs. This would enhance the accommodation of an active region of membrane within the resulting shortened BCD.

### QT interval and wavelength restitution analysis of data from multiple horses

**Fig 7:**
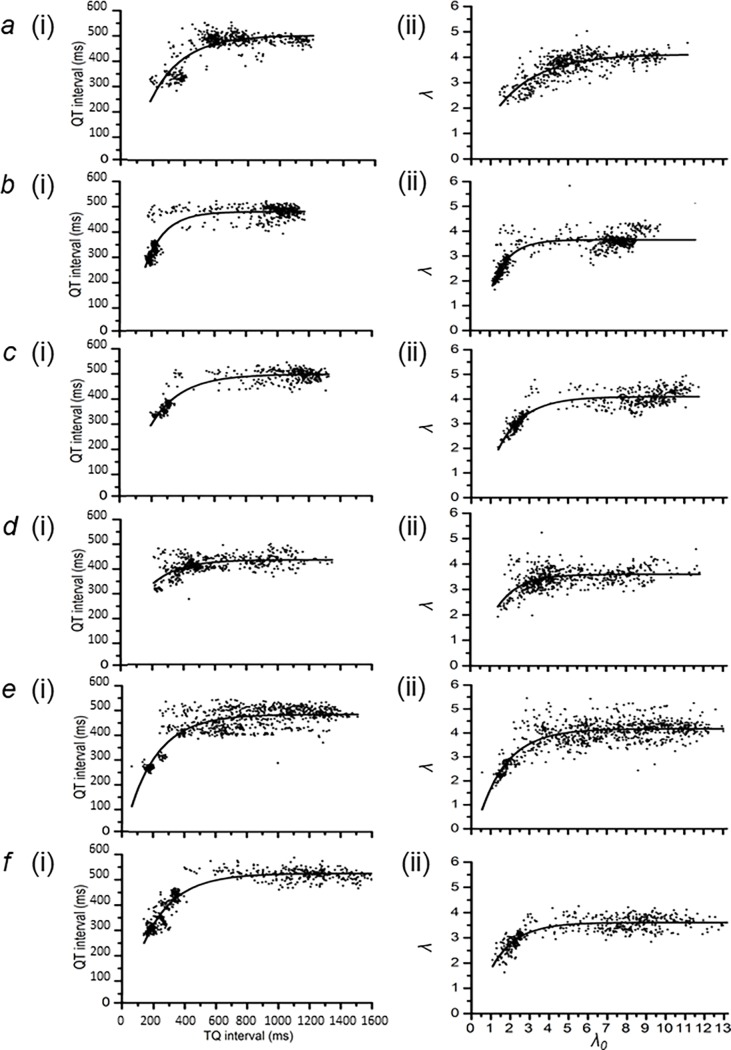
Comparison of electrocardiographic parameters at baseline and critical HRs. (a)–(f) Plots of QT vs. TQ interval ((i)) and lambda vs. lambda0 ((ii)), representing of action potential duration and wavelength restitution respectively, from the same 6 thoroughbred horses as in Fig 6. (a) Eq34 (a 2 year old male thoroughbred), (b) Eq39 (a 3 year old female thoroughbred), (c) Eq44 (a 4 year old female thoroughbred), (d) Eq52 (a 2 year old male thoroughbred), (e) Eq56 (a 2 year old male thoroughbred) and (f) Eq67 (a 3 year old male thoroughbred).

Fig 7 completes the analysis of results obtained from the same six horses ((a) to (f)) as Fig 6. It displays the outcomes of shows the results of (i) restitution analysis of QT intervals at different TQ intervals, derived from data of the kind illustrated in Fig 5. These are compared with (ii) a plot of *λ* against *λ0* from data shown in Fig 6. Both data sets were described by the function, *y* = *y*_0_ + [*A* × (1 − *e*^−*x*/*τ*^)], as adopted in previous reports [26, 27, 33]. In this equation, values of the ordinate and abscissa are denoted by *x* and *y* respectively. The least-squares fitting procedure determined the values for *y*_0_, *A* and *τ* for each data set. The constant *y*_0_ denotes the *y*-intercept of each fitted curve. *A* is a further constant that determines the maximum value of the term containing the exponential function. The time constant, *τ*, represents the steepness with which *y* declines at the lowest values of *x*.

This parameterization was then used to calculate the gradient of the curve at any given position on the x-axis. Thus, the slope of the fitting equation is given by the equation $\frac{dy}{dx}=\frac{A}{\tau }\times e^{-x/\tau }$. Restitution analysis indicates that the *x*-value at which d*y*/d*x* increases to unity value, where d*y*/d*x* = 1, corresponds to a transition from stable into unstable electrophysiological activity capable of generating sustained alternans leading to arrhythmia. This gives the equation *x*_*crit*_ = *τ* × *log_e_*(*τ*/*A*), where x_crit_ is the abscissa value corresponding to transformation into such instability. Employing this equation, QT interval restitution analysis in Fig 7i could then be used to obtain the corresponding critical TQ intervals. Similarly wavelength restitution in Fig 7ii provided the critical values for *λ*_0_, denoted as *λ*_*0crit*_. The corresponding critical ordinate values are then given by substituting x_crit_ in the original equation to give: $y_{crit}=y_{0}+A\times (1-e^{x_{crit}/\tau })$. The term y_crit_ is then either the critical QT interval or the critical *λ* (*λ*_*crit*_), as for QT interval and wavelength restitution respectively. In the QT interval restitution analysis, the critical value of the basic cycle length, BCL_crit_, could then readily be obtained as BCL is the sum of each pair of TQ and QT intervals, thus: BCL = QT interval + TQ interval. In wavelength restitution analysis, BCL_crit_ was obtainable from the plots of *λ*_*0*_ against BCL (Fig 6ii). In both analyses, the resulting value of BCL_crit_ was then used to calculate the value of the critical HR, HR_crit_.

Finally, it was possible to characterize electrocardiographic features of ECGs obtained at lowest HRs (HR_min_) observed in each experimental protocol. This corresponds to the maximum BCL observed, BCL_max_. The values of both the TQ interval, termed the maximum TQ interval, and *λ*_*0*_, termed *λ*_*0max*_, corresponding to the BCL_max_, could then be obtained from the highest x-values in Fig 7i and 7ii respectively. Their corresponding, maximum QT intervals and maximum *λ* (*λ*_*max*_*)*, is the sum of (A and y_0_) in Fig 7i and 7ii respectively. These features could then be compared to corresponding parameters at the HR_crit._

### Comparison of electrocardiographic parameters at baseline and critical HRs

Tables 1 and 2 displays these computed ECG features at the respective BCL_max_ and BCL_crit_ values, for all seven horses subjected to the detailed analysis, obtained from QT interval restitution (Table 1) and wavelength restitution (Table 2) respectively. Both tables summarize the empirical values of *y*_0_, *τ* and *A* of the fitted curve in each individual horse, the relevant derived parameters for each horse, and the overall (mean ± SEM) of each parameter for all the horses. Comparison of QT interval and the wavelength restitution analysis methods revealed that BCL_crit_ and HR_crit_ derived from the two methods were not statistically different (p > 0.05, n = 7). Yet, QT interval restitution analysis is based entirely on an analysis of AP recovery, whereas wavelength restitution analysis incorporates both CV and recovery properties. This concordance would attribute electrophysiological stability to the recovery as opposed to conduction properties of the AP. This was consistent with the observation in Fig 5B) that the velocity term expressed as 1/(QRS duration) showed variations at considerably lower TQ intervals than QT intervals.

**Table 1:** ECG characteristics at baseline and HR_crit_, from analysis of QT interval restitution.

	Restitution function parameters			ECG features at baseline				ECG features at critical conditions			
Horse	y_0_ (ms)	τ(ms^-1^)	A (ms)	HR_min_ (bpm)	BCL_max_ (ms)	Max. QT interval (ms)	Max TQ interval (ms)	HR_crit_ (bpm)	BCL_crit_ (ms)	Critical QT interval (ms)	Critical TQ interval (ms)
34	-162.4	201.0	666.1	35.4	1696.0	503.7	1210.0	110.4	543.6	302.8	240.8
39	-326.2	124.7	808.0	36.6	1640.0	481.8	1172.0	101.7	590.1	357.1	233.0
44	-80.5	191.9	579.6	32.9	1824.0	499.0	1326.0	115.5	519.3	307.2	212.1
46	86.4	233.5	377.3	39.2	1532.0	463.7	1114.0	175.3	342.3	230.2	112.1
52	105.8	172.0	323.7	34.0	1764.0	429.4	1346.0	163.9	366.2	257.4	108.7
56	-43.8	193.1	525.8	30.3	1982.0	482.0	1510.0	124.4	482.3	288.9	193.4
67	-60.7	196.3	578.6	22.3	2686.0	517.9	2188.0	112.4	533.8	321.6	212.2
Mean (±SEM)	-68.8 ± 55.7	187.5 ± 12.5	551.3 ± 62.4	32.9 ± 2.1	1874.9 ± 145.5	482.5 ± 11.1	1409.4 ± 138.9	129.1 ± 10.8	482.5 ± 35.4	295.0 ± 15.7	187.5 ± 20.7

**Table 2:** ECG characteristics at baseline and HR_crit_, from analysis of wavelength restitution.

	Restitution function parameters			ECG features at baseline				ECG features at critical conditions				
Horse	y_0_	τ	A	HR_min_ (bpm)	BCL_max_ (ms)	λ_max_	λ_0max_	HR_crit_ (bpm)	BCL_crit_ (ms)	λ_crit_	λ_0crit_
34	0.1	2.1	4.1	35.4	1696.0	4.1	11.2	133.1	450.8	2.0	1.4
39	-3.0	0.9	6.6	36.6	1640.0	3.7	11.6	103.1	582.0	2.8	1.8
44	-1.9	1.3	6.0	32.9	1824.0	4.1	11.8	104.2	575.5	2.7	2.0
46	-0.8	0.9	4.0	39.2	1532.0	3.3	7.5	138.8	432.4	2.4	1.4
52	-0.8	1.2	4.4	34.0	1764.0	3.6	11.8	115.1	521.4	2.4	1.5
56	-0.8	1.5	5.0	30.3	1982.0	4.2	13.0	123.8	484.5	2.7	1.8
67	-0.5	1.3	4.1	22.3	2686.0	3.6	16.3	103.6	579.4	2.3	1.5
Mean (±SEM)	-1.1 ± 0.4	1.3 ± 0.2	4.9 ± 0.4	32.9 ± 2.1	1874.9 ± 145.5	3.8 ± 0.1	11.9 ± 1.0	117.4 ± 5.6	518.0 ± 24.0	2.5 ± 0.1	1.6 ± 0.1

Electrophysiological features at baseline and close to the limits of stable activity summarized in the above Tables provide the basis for the comparisons in Tables 3 and 4. These summarize the associated increase in HR and reduction in BCL, QT interval, and TQ interval, and the corresponding fall in *λ*, and *λ*_*0*_, expressed both as absolute values and percentages.

**Table 3:** Comparison of results of QT interval restitution against electrocardiographic parameters at baseline HR.

	Absolute change in value				% change in value			
Horse	ΔHR (bpm)	ΔBCL (ms)	Δ(QT interval) (ms)	Δ(TQ interval) (ms)	% change in HR	% change in BCL	% change in QT interval	%change in TQ interval
34	75.0	1152.4	201.0	969.2	212.0	67.9	39.9	80.1
39	65.1	1049.9	124.7	939.0	177.9	64.0	25.9	80.1
44	82.6	1304.7	191.9	1113.9	251.2	71.5	38.4	84.0
46	136.1	1189.7	233.5	1001.9	347.6	77.7	50.4	89.9
52	129.8	1397.8	172.0	1237.3	381.8	79.2	40.1	91.9
56	94.1	1499.7	193.1	1316.6	310.9	75.7	40.1	87.2
67	90.1	2152.2	196.3	1975.8	403.2	80.1	37.9	90.3
Mean (±SEM)	96.1± 10.2	1392.4 ± 139.1	187.5 ± 12.5	1221.9± 136.4	297.8 ± 32.6	73.7 ± 2.3	38.9 ± 2.7	86.2 ± 1.8

**Table 4:** Comparison of results of wavelength restitution against electrocardiographic parameters at baseline HR.

	Absolute change in value				% change in value			
Horse	ΔHR (bpm)	ΔBCL (ms)	Δ*λ*	Δ*λ*_*0*_	% change in HR	% change in BCL	% change in *λ*	%change in *λ*_*0*_
34	97.7	1245.2	2.1	9.8	276.2	73.4	51.3	87.7
39	66.5	1058.0	0.9	9.8	181.8	64.5	24.4	84.6
44	71.4	1248.5	1.3	9.8	216.9	68.4	32.9	82.9
46	99.6	1099.6	0.9	6.1	254.3	71.8	27.5	82.0
52	81.1	1242.6	1.2	10.3	238.3	70.4	32.0	87.0
56	93.6	1497.5	1.5	11.2	309.1	75.6	35.0	86.3
67	81.2	2106.6	1.3	14.8	363.6	78.4	35.4	90.9
Mean (±SEM)	84.4 ± 4.9	1356.8 ± 135.8	1.3 ± 0.2	10.3 ± 1.0	262.9 ± 22.8	71.8 ± 1.7	34.1 ± 3.3	85.9 ±1.2

On average, HR_crit_ was attained at a rate higher, by 96.1 ± 10.2 bpm and 84.4 ± 4.9 bpm, than HR_min_, as derived from QT interval and wavelength restitution analysis respectively. This corresponds to a ~3 and ~2.5 fold increase in HR. Accordingly, with this increase in HR, the percentage reductions in BCL, QT interval, and TQ interval was 73.7 ± 2.3%, 38.9 ± 2.7%, and 86.2 ± 1.8% respectively, and this was similar to that observed for BCL, *λ* and *λ*_*0*_ calculated from wavelength restitution analysis. These were 71.8 ± 1.7%, 34.1 ± 3.3%, and 85.9 ± 1.2% respectively.

With the increase in HR, there were reductions in both the temporal (TQ interval) and spatial (*λ*_*0*_) separation between successive APs. However, this was ameliorated by the marked reductions in temporal (QT interval) and spatial (*λ*) extent of the APs themselves, reflecting a marked capacity for the equine heart for physiological adjustments of excitable properties with increased HRs. Comparison of Tables 3 and 4 with Fig 7 demonstrate that this accommodation occurred at the lower range of x-values, where the y-values are decreasing from the plateau component of the restitution curves.

## Discussion

The repetitive activity of the heart consists of a sequence of action potentials (APs) separated by recovery periods, diastolic intervals (DIs), when the ion channel underlying this electrical activity returns to the resting state. The maintenance of this sequence ensures orderly activation of the next AP. A disruption of this regular activity, arrhythmia, can occur at high heart rates, such as during exercise, where the diminished DIs that allow for AP accommodation could compromise recovery. This could in turn compromise activation of the subsequent AP and its timecourse. Nevertheless, in vivo cardiac activity involves sympathetic nervous system activation at higher heart rates that shortens the AP durations (APDs), giving a longer time for recovery, and therefore allows a stable higher heart rate. However, on rare occasions, exercise can be associated with instable adaptation and major cardiac arrhythmic events. Restitution analysis indicates that this instability can occur when the slope of APD against DI exceeds one, causing a substrate for arrhythmogenesis.

However, previous restitution analyses often involved isolated, and therefore denervated, Langendorff perfused, hearts in which changes in HR were achieved by imposed stimulation and not in the normal physiological course of activity [26–28]. This limits the translatability of these findings to understanding the pathophysiological basis of sudden cardiac death in the human athlete. The present work adapts the restitution experimental analysis from invasive in vitro studies to non-invasive in vivo study for the first time in the equine model. The protocol used here thus emulated incremental pacing protocols that have previously been applied in studies of cardiac function in vitro in other animal species. The equine model thus gives a wide range of basic cycle lengths (BCLs) that could allow it to become a model for the human athlete.

We thus report a detailed telemetric electrocardiographic (ECG) analysis to explore normal action potential (AP) propagation and waveform in healthy racing Thoroughbred horses taken through period of acceleration from walk to canter. We explored the electrophysiological capacity for the heart in the intact horse to function through its normal range of low, baseline through high, exercising heart rates. ECG records were obtained with atraumatically fitted recording equipment [18]. The exercise period yielded a wide range of BCLs from which a heart rate (HR) could be derived from individual BCLs. Our analysis assumed established physiological interpretations of the particular components of our observed ECG records [36], and used conventions for their measurement accepted in and therefore translatable to human clinical practice [37].

The ECGs first confirmed that all the horses studied normally showed a regular pattern of sinus rhythm followed by normal QRST complexes, with narrow QRS complexes and stable QT intervals at all HRs, reflecting normal and regular sino-atrial, atrial and ventricular activity [39, 40]. A subgroup consisting of 7 of these were amenable to full quantitative analysis. These were could be studied at closely incremented frequency intervals over a wide range (22–181 bpm). Thus, the baseline equine HRs were lower than the corresponding normal resting human HRs (~70 bpm). Typically, athletic horses can show resting HRs as low as ~30 bpm and slightly higher baseline HRs noted in these individuals would likely be associated with excitement as the recordings were obtained while horses were being prepared for exercise testing, including insertion of an intra-nasal endoscope. In the general population, a HR of ~20 bpm is associated with underlying pathology, such as 3^rd^ degree heart block.

Secondly, we could assess for the presence or absence of QT alternans in equine hearts. This demonstrated transient rather than persistent episodes of such alternans. In human clinical practice, such alternans has been reported at high HRs or in the presence of cardiac pathology. If persistent, it is known to presage potentially fatal arrhythmia [26, 27, 29, 41–47]. A total number of 64 episodes of alternans were seen across all seven horses. However, these were transient rather than sustained and could occur anywhere within the range of HRs that were studied rather than the highest rates, and therefore would not be expected to be associated with major arrhythmias. Thirdly, we could reconstruct ventricular action potential (AP) characteristics from our quantifications of the QRST complexes using established physiological interpretations of the different components of the ECG signal. Thus, estimates of BCL, ventricular conduction velocities, APD and DI were derived from RR intervals, QRS durations, QT interval and TQ interval respectively [48]. As this was the first time such an exploration on equine ECGs were undertaken, only ECG complexes that were clearly discernible were analysed. This led to exclusion of ECG complexes at maximal heart rates. ECG complexes at this range typically have multiple movement artefacts thus preventing the reliable detection of the relevant peaks and troughs. Despite this, the study was able to analyse a wide range of incremental heart rate allowing for restitution analysis to be performed.

Finally, we could use these readouts in quantitative, restitution, analyses used to predict arrhythmic substrate at high HRs that had been introduced in previous in vitro studies in other animal species [26–32, 49–51]. The steps in these analyses are detailed in a single example; this is followed by statistical analysis of the results for all the horses studied. These derived the temporal properties of AP recovery in the form of action potential durations, (APDs), at different diastolic intervals (DIs), from QT interval and TQ interval respectively. Variations in these parameters with basic cycle length (BCL) reflecting varying HRs were then examined. A similar analysis had been used to explore arrhythmic tendency associated with long QT syndrome [28, 52] by measuring the decline of APD with decreasing DI at different heart rates from a plateau value. This led to a more complete analysis, which has been employed in analysis of arrhythmic substrates in models of the Brugada Syndrome. This first plotted QRS durations at different RR and TQ intervals. This demonstrated an increase in conduction velocity at high HRs. It then combined AP recovery and conduction terms to give wavelength terms representing electrical activity (*λ*) and subsequent periods of rest (*λ*_*0*_) [27]. The *λ* term represents the length of tissue depolarised by the AP wavefront; the *λ*_*0*_ term is the length of repolarised tissue trailing behind the depolarised tissue. The *λ* term was represented by the quotient QT/QRS, and the *λ*_*0*_ term by TQ/QRS. Plots of *λ* against *λ*_*0*_ provided a spatial means of predicting arrhythmic substrate.

The above analyses yielded functions in agreement with findings obtained on earlier occasions. Thus, both plots QT interval against TQ interval, and of *λ* against *λ*_0_, followed the function *y* = *y*_0_ + [*A* × (1 − e^−x/τ^)]. Thus, QT interval and *λ* assumed a plateau at the lower HRs and declined only at the highest HRs. This would predict an accommodation of QT interval and *λ* at high HRs. This would in turn mitigate the reduction in TQ interval and *λ*_*0*_ with increasing HR. This would enhance the time permitted for tissue to recover from refractoriness following excitation and optimizes diastolic filling of the ventricular chambers. Furthermore, either analysis could yield critical values of APD_crit_, DI_crit_ or of *λ*_crit_, *λ*_0crit_, when the plots attained unity slope. These in turn yielded the critical HR (HR_crit_) at which arrhythmic substrate was expected. The analyses extended from minimum values of equine HRs (HR_min_ = 32.9 ± 2.1 (n = 7) bpm). HRs could increase to statistically indistinguishable levels of 129.1 ± 10.8 (n = 7) or 117.4 ± 5.6 (n = 7) bpm, i.e. ~3 to 2.5 times the baseline rate, before reaching HR_crit_, as measured from QT interval or *λ* respectively. This reflects a large statistically similar range of permissible HRs (of 96.1 ± 10.2 (n = 7) and 84.4 ± 4.9 (n = 7) bpm) from measurements of QT interval and *λ* respectively). This was achieved by shortening QT interval to 60% of its maximum value from 482.5 ± 11.1 ms to 295.0 ± 15.7 ms, or by shortening *λ* to 66% of its maximum value from 3.8 ± 0.1 to 2.5 ± 0.1.

## Conclusion

The present in vivo study provides a physiological basis for the large range between HR_min_ and HR_crit_ in the absence of sustained alternans throughout this study. This explains why horses are capable of showing high HRs without compromising their electrophysiological stability. This ability of a horse’s heart to increase HR allows the heart to meet the metabolic demands during exercise and racing. The present in vivo approach deduced the relevant AP parameters from strategic ECG parameters. This study suggests a strong potential of the equine athlete being used to model the cardiac electrophysiology of the human athlete and future studies should aim to validate this further.

## Supporting information

S1 Dataset Excel file of dataset of electrocardiographic intervals in all horses. (XLSX)
